# Cathelicidin-like Helminth Defence Molecules (HDMs): Absence of Cytotoxic, Anti-microbial and Anti-protozoan Activities Imply a Specific Adaptation to Immune Modulation

**DOI:** 10.1371/journal.pntd.0002307

**Published:** 2013-07-11

**Authors:** Karine Thivierge, Sophie Cotton, Deborah A. Schaefer, Michael W. Riggs, Joyce To, Maria E. Lund, Mark W. Robinson, John P. Dalton, Sheila M. Donnelly

**Affiliations:** 1 Institute of Parasitology, McGill University, Sainte-Anne-de-Bellevue, Quebec, Canada; 2 Laboratoire de Santé Publique du Québec, Institut National de Santé Publique du Québec, Sainte-Anne-de-Bellevue, Quebec, Canada; 3 Department of Veterinary Science and Microbiology, University of Arizona, Tucson, Arizona, United States of America; 4 The ithree Institute, University of Technology Sydney (UTS), Sydney, Australia; 5 School of Biological Sciences, Queen's University Belfast, Medical Biology Centre, Belfast, Northern Ireland; Uniformed Services University of the Health Sciences, United States of America

## Abstract

Host defence peptides (HDPs) are expressed throughout the animal and plant kingdoms. They have multifunctional roles in the defence against infectious agents of mammals, possessing both bactericidal and immune-modulatory activities. We have identified a novel family of molecules secreted by helminth parasites (helminth defence molecules; HDMs) that exhibit similar structural and biochemical characteristics to the HDPs. Here, we have analyzed the functional activities of four HDMs derived from *Schistosoma mansoni* and *Fasciola hepatica* and compared them to human, mouse, bovine and sheep HDPs. Unlike the mammalian HDPs the helminth-derived HDMs show no antimicrobial activity and are non-cytotoxic to mammalian cells (macrophages and red blood cells). However, both the mammalian- and helminth-derived peptides suppress the activation of macrophages by microbial stimuli and alter the response of B cells to cytokine stimulation. Therefore, we hypothesise that HDMs represent a novel family of HDPs that evolved to regulate the immune responses of their mammalian hosts by retaining potent immune modulatory properties without causing deleterious cytotoxic effects.

## Author Summary

In mammals, secreted host defence peptides (HDPs) protect against a wide range of infectious pathogens. They also perform a range of immune modulatory functions which regulate the immune response to pathogens, ensuring that the protective inflammatory response is not exacerbated and that post-infection repair mechanisms are initiated. We identified a novel family of molecules secreted by medically-important helminth pathogens (termed helminth defence molecules; HDMs) that exhibit striking structural and biochemical similarities to the HDPs. To further investigate the extent of this similarity, we have performed a comparative functional study between several well characterized, anti-microbial, mammalian HDPs and a series of parasite-derived peptides. The parasite HDMs displayed immune modulatory properties that were similar to their HDP homologs in mammals, but possessed no antimicrobial or cytotoxic activity. We propose that HDMs of these helminth pathogens underwent specific adaptation, losing their anti-microbial activity but retaining their ability to regulate the immune responses of their mammalian hosts. This absence of cytotoxicity and retention of immune-modulatory activity offers an opportunity to design novel immunotherapeutics derived from the HDMs which could be used to combat destructive inflammatory responses associated with microbial infection and immune-related disorders.

## Introduction

Host defence peptides (HDPs) are found in all living organisms and play a pivotal role as effector components of the innate immune system [1], [2]. They act as the first line of defence against pathogenic assaults from bacteria, fungi, eukaryotic parasites and viruses [3]–[5]. A range of HDPs with varied sequence lengths, structures and activities have been characterized [6] and since sequence identity between them is often very poor, their classification is based largely on homologous secondary structures. The two predominant HDP groups found in nature are the cathelicidins, characterized by α-helical secondary structure, and the defensins, which contain β-sheets stabilized by intra-molecular disulfide bridges [7]–[9]. Despite the diversity in their sequences and structures, HDPs are typically small amphipathic peptides (12–50 amino acids) with a net positive charge (+2 to +9) and consist of at least 50% hydrophobic amino acids [10]. These biochemical properties are central to the HDPs antimicrobial function by allowing their interaction with, and disruption of, negatively charged bacterial membranes [10].

The contribution of mammalian HDPs to the innate immune response extends beyond direct bacterial killing. The elevated expression of HDPs in response to damage (injury or infection) has led to the suggestion that mammals utilize these peptides as ‘alarmins’ to activate the mobilization of a comprehensive immune response [11]. Besides their antimicrobial activity, HDPs function as potent immune regulators, selectively altering host gene expression, inducing chemokine production, inhibiting bacterial- or hyaluronan-induced pro-inflammatory cytokine production, promoting wound healing and modulating T and B cell function [reviewed in [12]–[14]. The net result of these activities is a balance between pro- and anti-inflammatory immune responses which prevents an exacerbated inflammatory response while concurrently stimulating the resolution of infection and repair of damaged epithelia.

The immune response elicited by helminth (worm) parasites is akin to the innate immune response to tissue injury and wound healing [15], [16]. Typically, this consists of a suppression of classical pro-inflammatory responses and the induction of anti-inflammatory regulatory Th2 type immune responses. While classical Th1-associated inflammatory mediators can provide protection from helminths [17], there is a substantial cost in collateral damage to host tissue [15], [18]. In addition, due to their migration and feeding activities, helminth parasites cause considerable local tissue damage. Therefore, it has been proposed that on exposure to helminths, the most beneficial outcome is to shut down a destructive Th1-type response in favour of a Th2 response that rapidly and effectively heals tissue [15], [17], [18]. Ultimately this means that the parasite is tolerated by the host, remaining *in situ* for many years and thus successfully completes its lifecycle.

Some advances have been made in identifying the signalling molecules that initiate helminth-associated Th2 responses. Many of these (such as IL-33 and Thymic stromal lymphopoietin (TSLP)) are thought to be released by epithelial cells damaged by migrating parasites [15], [19]. However, a number of helminth-derived products have also been shown to modulate the function of innate immune cells and thus are potentially instrumental in the initiation of Th2 immune responses [19], [20]. We have previously shown that a cysteine protease secreted by the trematode helminth *Fasciola hepatica* prevented the induction of pro-inflammatory macrophages and dendritic cells [21], [22]. In addition, peroxiredoxin, also secreted by *F. hepatica*, promoted the development of Th2 host immune responses [23], [24]. Importantly, homologues of these proteins are found in other medically-important trematode parasites which we have suggested reveals a common mechanism of immune-modulation employed by this class of pathogen.

As part of our on-going analysis of the secretome of *F. hepatica* we recently discovered a novel 8 kDa protein. On analysis, this protein shared structural and biochemical similarities to mammalian cathelicidins and was therefore termed *F. hepatica* Helminth Defence Molecule 1 (FhHDM-1) [25]. Like the human cathelicidin LL-37 precursor CAP-18, FhHDM-1 is proteolytically processed (by a parasitic endopeptidase, cathepsin L1) to release a 34-residue C-terminal peptide previously named FhHDM-1 p2 [25]. This peptide adopts an amphipathic helix structure and, like LL-37, can bind to *Escherichia coli* lipopolysaccharide (LPS) to prevent its interaction with the toll-like receptor (TLR) 4/MD2/CD14 complex on macrophages. Hence we proposed that *F. hepatica* utilized FhHDM-1 as a molecular mimic of mammalian cathelicidin-like HDPs as a means of controlling host innate immune responses [25].

Phylogenetic analysis showed that FhHDM-1 is a member of a family of HDMs conserved throughout several major animal and human trematodes such as *Schistosoma*, *Fasciola*, *Opisthorchis*, *Clonorchis* and *Paragonimus* species [25]. Importantly, all HDM molecules in this family have preserved the C-terminal amphipathic helix. Here, we have performed a comparative functional study between several anti-microbial HDPs derived from well-characterized mammalian cathelicidins and parasite-derived peptides. For the helminth-derived peptides we selected FhHDM-1p2 and two homologs derived from *Schistosoma mansoni* which we term *S. mansoni* HDM-1 (SmHDM-1p146) and HDM-2 (SmHDM-2p58). In addition, we included a peptide derived from a previously characterized secretory molecule of *S. mansoni*, termed Sm16-p73 [26], which our phylogenetic studies suggest is a divergent member of the HDM superfamily [25]. We show that, in contrast to the mammalian HDPs (LL-37, CRAMP, BMAP-28 and SMAP-29), the trematode-derived HDMs are not cytotoxic or bactericidal. However, like the mammalian HDPs, the trematode HDMs suppress the activation of macrophages by microbial stimuli and alter the isotype of immunoglobulin secreted by B cells. We propose that HDMs represent a novel family of HDPs that have undergone specific adaptation to retain potent immune modulatory properties in the absence of deleterious cytotoxic effects and are exploited by helminth pathogens to regulate the immune responses of their mammalian hosts.

## Methods

### Synthetic mammalian and trematode peptides and their biochemical properties

Four synthetic cathelicidin-derived peptides from diverse mammalian species were used: SMAP-29 from sheep [27], [28], CRAMP from mice [29], LL-37 from human [30], and BMAP-28 from cattle [31]. The 34-residue C-terminal FhHDM-1 peptide, termed FhHDM-1p2, has been previously described [25]. Sm16-p73 peptide is 35 residues in length, corresponding to residues 73–107 of the full-length protein *S. mansoni* Sm16 (GenBank accession number: AAD26122.1). SmHDM-1p146 is 35 residues in length and corresponds to residues 146–180 of the full length protein (GenBank accession number XP_002580563.1). Finally, SmHDM-2p58 is a 32 residue peptide that corresponds to residues 58–98 from the full length protein (GenBank accession number: XP_002576627.1). All mammalian and trematode peptides were synthesized by GenScript (NJ, USA) and supplied endotoxin-free. The single letter code sequence of each peptide is shown in Table 1.

**Table 1:** Mammalian- and Helminth-derived peptides used in this study and their biochemical characteristics.

Peptides	Source	Sequence	Residues	Net Charge^1^	Boman index kcal/mol^2^	Hydrophobic ratio (%)^3^	hydrophobic residues on same surface
FhHDM-1p2	*F. hepatica*	AMAYLAKDNLGEKITEVITILLNRLTDRLEKYAG	34	0	1.34	44	14
Sm16-p73	*S. mansoni*	MDKYIRKEDLGMKMLDVAKILGRRIEKRMEYIAKK	35	+5	2.62	40	8
SmHDM-1p146	*S. mansoni*	IDTYSRKDDLDKKLLEVAKIIGRRLEKRHEYLAMK	35	+3	3.11	34	8
SmHDM-2p58	*S. mansoni*	IRKYLEEDNLGEKLAAVVSIYVKRLNKRLDMR	32	+3	2.6	40	8
LL-37	Human	LLGDFFRKSKEKIGKEFKRIVQRIKDFLRNLVPRTES	37	+6	2.99	35	10
CRAMP	Mouse	ISRLAGLLRKGGEKIGEKLKKIGQKIKNFFQKLVPQPE	38	+7	1.59	34	10
SMAP-29	Sheep	RGLRRLGRKIAHGVKKYGPTVLRIIRIA	28	+9	2.27	39	11
BMAP-28	Bovine	GGLRSLGRKILRAWKKYGPIIVPIIRIG	28	+7	0.81	42	9

1 Charge of peptide at pH 7.0.

2 The Boman index is an estimate of the potential of peptides to bind to other proteins, such as different receptors, and is defined as the sum of the free energies of the amino acid residues side chains divided by the total number of amino acid [48].

3 Ratio of hydrophobic residues/total number of residues (%).

The biochemical characteristics for each of the HDMs and HDPs were calculated using tools available from the Antimicrobial Peptide Database (http://aps.unmc.edu/AP/main.php) [32] and are presented in Table 1. Predicted properties were total net charge, Boman index, hydrophobic ratio and total hydrophobic residues on the same hydrophobic surface of the alpha helix.

### HDM sequence analysis

We have previously shown, using circular dichroism (CD) spectroscopy, that FhHDM-1 has the propensity to adopt alpha-helical structure in solution [25]. To assess whether HDMs from the related trematode parasite *S. mansoni* also form alpha helices, secondary structure prediction was performed using JPred 3 ([33]; http://www.compbio.dundee.ac.uk/www-jpred/). Specific regions predicted to form alpha helices were then subjected to helical wheel analysis using Heliquest ([34]; http://heliquest.ipmc.cnrs.fr/cgi-bin/ComputParams.py) to identify those with distinct hydrophobic faces; i.e. are amphipathic. The atomic structures of the vertebrate HDPs LL-37 (PDB ID: 2K6O) and BMAP-28 (PDB ID: 2KET) were visualised for comparison using the PyMOL Molecular Graphics System, Version 1.5.0.4 Schrödinger, LLC. (http://pymol.org/).

### Bacterial lipopolysaccharide binding assay

Lipopolysaccharide (LPS) binding was performed using a quantitative chromogenic *Limulus* amoebocyte assay (Chromo-LAL assay; Associates of Cape Cod Incorporated) following manufacturer's recommendations. Assays were performed in flat-bottom endotoxin- and glucan-free 96-well plates (Associates of Cape Cod Incorporated). Stock solutions of each peptide were prepared in endotoxin-free water (80 µg/ml) and diluted to a final concentration of 250 pmol/ml. In the first step, 25 µl of peptide solution was mixed with 25 µl of a solution containing 1 endotoxin U/ml of *Escherichia coli* O113:H10 LPS and incubated for 30 min at 37°C to allow peptide and LPS binding to occur. The second step involved the addition of 50 µl of the chromo-LAL reagent. The liberation of ρ-nitroaniline was monitored every 60 sec at 405 nm with a Synergy H1 hybrid reader (Biotek) while the temperature was maintained at 37°C. Each peptide concentration was also incubated with 25 µl of LPS-free water as a control to determine if the peptide itself could activate the Chromo-LAL assay. The experiment was conducted twice in triplicate. Standard deviation was calculated from these six replicates.

### Antimicrobial assays

The minimal inhibitory concentration (MIC) of each peptide against various bacteria was determined using a standardized dilution method according to NCSLA guidelines [35]. Overnight colonies of *E. coli*, *Pseudomonas aeruginosa*, *Salmonella typhimurium*, *Staphylococcus epidermis* and *Staphylococcus aureus*, were suspended to a turbidity of 0.5 OD units and further diluted in Mueller-Hinton broth (MHB). For determination of MIC, peptides were prepared in an acetic acid/BSA solution and used in graded concentrations (0, 1, 2, 4, 8, 16, 32, 64, and 128 µg/ml) from a stock solution. Ten microliters of each concentration was added to each corresponding well of a 96-well polypropylene microtiter plate and 1×10^5^ bacteria in the volume of 90 µL. The plate was incubated at 37°C for 16 h and then read at 600 nm with a Synergy H1 hybrid reader (Biotek).

### Haemolytic activity

The peptide's haemolytic activities were determined using human red blood cells (RBCs; Research Blood Components, LLC) in 96-well polypropylene microtiter plates. One hundred µl of 0.5% RBC suspension was added to an equal volume of a peptide (8–256 µg/ml). After 1 h at 37°C, plates were centrifuged at 1420×g for 5 min and the optical density of the supernatant was measured at 414 nm with a Synergy H1 hybrid reader (Biotek). Values for 0% and 100% lysis were obtained by adding PBS or Triton X-100 (1%; final concentration) to RBCs, respectively. All assays were performed in triplicate and the values of percent lysis were within a 1% standard deviation range.

### Measurement of cellular pore formation

RAW 264.7 murine macrophages (5×10^5^ cells) were incubated in the presence of the fluorescent dye TO-PRO (Life Technologies) for 60 sec. After the peptides (50 µM) were added to the culture media the uptake of dye was measured for 360 sec by flow cytometry.

### Cytotoxicity assay

RAW 264.7 murine macrophages (1×10^6^cells) were incubated with a range of concentrations (2.5–50 µM) of peptides for 1 h at 37°C. The culture supernatants were collected and assayed for LDH activity with the CytoTox LDH release kit (Promega) according to the manufacturer's instructions. The amount of LDH released is expressed as a percentage of the total amount of LDH released from cells treated with lysis buffer (regarded as 100% cytotoxicity).

### Effect of peptides on *Cryptosporidium spp.* sporozoite viability

Oocysts of the Iowa *C. parvum* isolate [36] were propagated in experimentally infected newborn *Cryptosporidium*–free Holstein bull calves to obtain parasite material for study as previously described [37], [38]. Oocysts were isolated by sucrose density gradient centrifugation, stored in 2.5% (W/V) potassium dichromate (4°C) and used within 6 weeks of isolation [39]. Oocysts of the TU502 *C. hominis* isolate [40], [41] were propagated in gnotobiotic piglets and isolated from feces at Tufts University [42] and used within 4 weeks of isolation. Prior to excystation, oocysts were treated with hypochlorite [37]. *In vitro* excystation (37°C, 0.15% [W/V] taurocholate, 2 h) of oocysts used for all experiments was ≥90%. Sporozoites were isolated from excysted oocyst preparations by passage through a polycarbonate filter (2.0 µm pore size; Poretics, Livermore, California) and used immediately.

Sporozoite viability after incubation with peptides was assessed using fluorescein diacetate (FDA) and propidium iodide (PI) with modification [43]. In brief, freshly excysted sporozoites were incubated (15 min, 37°C) in minimal essential medium (MEM) containing individual peptides (2.5, 0.25, 0.025 µM) or in MEM alone (n = 3). Peptide concentrations were selected based largely on studies by our group and others evaluating the effects of various antimicrobial peptides on *C. parvum* viability [44]–[47]. Heat-killed (20 sec, 100°C) sporozoites were used as a control. FDA (8 mg/ml final concentration) and PI (3 mg/ml final concentration) were then added to the sporozoite preparations and incubated further (5 min, 21°C). A minimum of 100 sporozoites were then examined by epifluorescence microscopy for each preparation, and the percent viability was determined. Percent reduction of viability was calculated as ([MEM-treated sporozoite viability−peptide-treated sporozoite viability]÷MEM treated sporozoite viability)×100. The mean values for test and control preparations were examined for significant differences using Student's *t*-test.

### Purification and activation of bone marrow-derived murine macrophages

CD11b^+^F4/80^+^ macrophages were derived (99% purity) from the bone marrow of BALB/c mice by culturing with M-CSF (ebioscience) over 6 days and then seeded in RPMI (with 10% FBS v/v) at a concentration of 1×10^5^ cells/ml. These cells were incubated with peptides (0.5 µM–5 µM) for 1 h at 37°C, in the absence of mCSF. Following two washes with ice-cold PBS, cells were incubated with a combination of *E. coli* LPS (10 ng/ml; Sigma) and IFNγ (10 ng/ml; BD Pharmingen) overnight. Supernatants were then collected and the amount of TNF measured by ELISA according to the manufacturer's instructions (BD Pharmingen).

### Purification and activation of murine B cells

B cells were isolated from the spleens of BALB/c mice by negative selection using a B cell isolation kit containing biotin-conjugated mAbs to CD43, CD4, and Ter-119 (Miltenyi Biotec) and then seeded at a concentration of 1×10^6^ cells/ml in RPMI (with 10% FBS v/v). The cells were treated with a range of concentrations of peptides (0.5 µM–5 µM) for 1 h at 37°C. After washing, the B cells were incubated with a combination of either *E. coli* LPS (10 µg/ml; Sigma) and IL-4 (10 ng/ml; BD Pharmingen) or *E. coli* LPS (10 µg/ml) and IFNγ (200 ng/ml; BD Pharmingen) for six days. Supernatants were then collected and the amount of IgG1 or IgG2a measured by ELISA (Sigma).

### Statistical analyses

Statistical comparisons were performed with Prism 4.0 Software (Graph- Pad), using two-tailed Student's *t* test for comparisons of two data sets, and ANOVA for multiple comparisons. Statistically significant differences were determined by a *p* value of *<0.05, **<0.01, ***<0.001.

## Results

### HDMs, like mammalian HDPs, are cationic, have a high percentage of hydrophobic amino acids and form amphipathic structures

The biochemical properties for each of the HDMs and HDPs are presented in Table 1. The cathelicidins are known to be highly cationic peptides. Except for the FhHDM-1p2 peptide, which has a net charge of 0, all the peptides in the present study have a net positive charge (+3 to +9) with a percentage of hydrophobic residues ranging from 34 to 44. The Boman index is an estimated potential of peptides to bind to other proteins. For this index, a low value (≤1) suggests that a peptide has more antibacterial activity, whereas values ranging from 2.5–3.0 indicate that a peptide is multifunctional with hormone-like activities [48]. While BMAP-28 has a low Boman index (0.81) which correlates to its high antimicrobial activity, the Boman indices for the other peptides range from 1.34–3.11. Overall, there is no striking difference between the biochemical properties of HDMs and HDPs.

**Figure 1:**
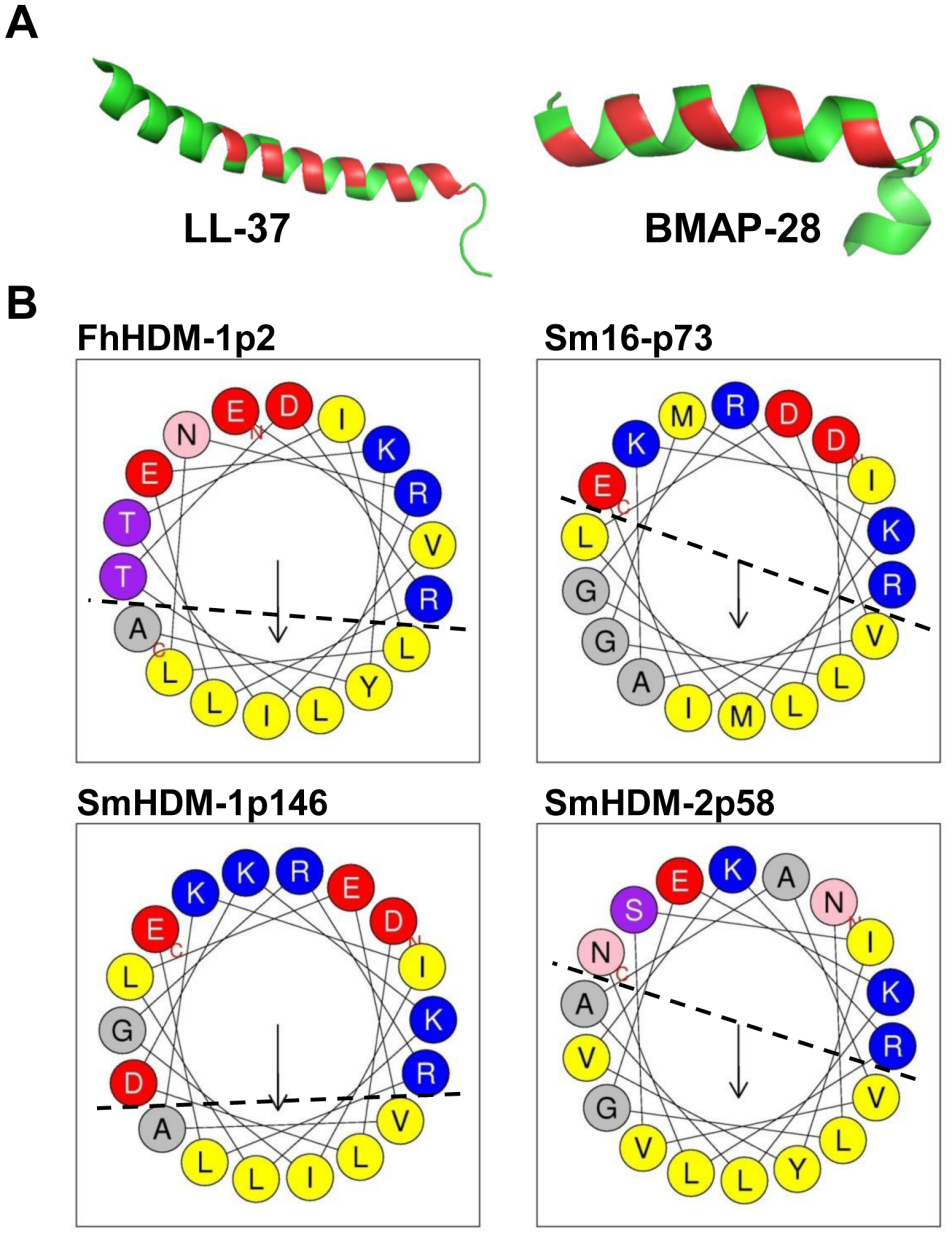
Mammalian and helminth cathelicidin-like peptides share homologous secondary structure. (A) Most HDPs, including those used in the present study, form amphipathic helices. Here, the atomic structures of LL-37 (PDB ID: 2K6O) and BMAP-28 (PDB ID: 2KET) are shown as green ribbon with the residues comprising the hydrophobic face of the molecules coloured red. Structures were generated using Pymol (http://www.pymol.org). (B) Helical wheel analysis shows that peptides derived from FhHDM-1p2 (residues 14–34), Sm16-p73, SmHDM-1p146 and SmHDM-2p58 (residues 9–26 for each) also form distinct amphipathic structures.

The atomic structures of the vertebrate HDPs LL-37, CRAMP, BMAP-28 and SMAP-29 have been previously solved and determined that each form an amphipathic helix [31], [49]–[51] (figure 1). The structures of LL-37 and BMAP-28 are shown in figure 1A as representative for this group of peptides. Secondary structure prediction of the parasite-derived peptides FhHDM-1p2, SmHDM-1p146, SmHDM-2p58 and Sm16-p73 revealed that all possessed regions likely to form alpha helices. Furthermore, helical wheel analysis showed that each molecule contained an alpha helix (32–35 amino acids) toward the C-terminal that was distinctly amphipathic. The number of residues forming the hydrophobic face of the parasite molecules ranged from 6–9 (figure 1B). Thus, like their vertebrate HDP counterparts, the secreted helminth parasite molecules also form distinct amphipathic helices.

### Helminth defence molecules variably bind LPS

Mammalian HDPs have the ability to bind to and thus neutralize the bacterial endotoxin LPS [52]–[57]. The capacity of different mammalian and helminth peptides to bind LPS from *E. coli* O113:H10 was compared using the chromogenic *Limulus* amoebocyte assay (Chromo-LAL). The *Limulus* amoebocyte lysate contains enzymes that are activated in a cascade of reactions in the presence of LPS [58]–[60]. The final enzyme in the series splits the chromophore, ρ-nitroaniline (ρNA), from the chromogenic substrate, generating a yellow color. The amount of ρNA released is proportional to the amount of free LPS present in the system.

**Figure 2:**
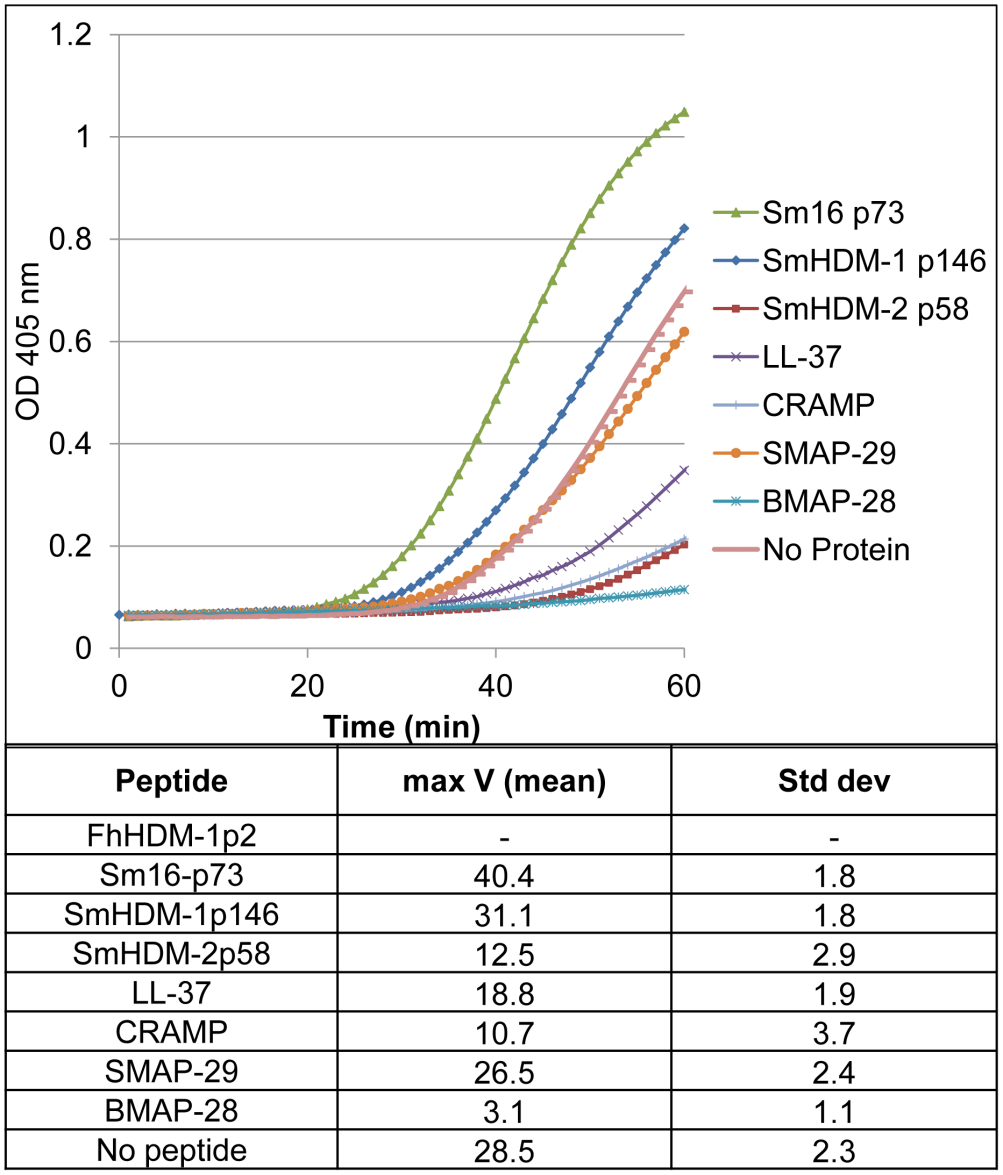
Mammalian and helminth cathelicidin-like peptides display varied LPS binding capacity. 25 µl of peptide (250 pmol/ml) was incubated with 25 µl of LPS from *E. coli* O113:H10 (1 EU/ml) for 30 min at 37°C to allow the interaction between both molecules. Chromo-LAL reagent was then added and the liberation of ρ-nitroaniline was monitored every 60 sec at 405 nm. The top panel is a representative of the curves obtained for each peptide. The data presented in the Table is the mean of three replicates from two separate experiments. The extent of binding of peptide to LPS prior to adding the mixture to the Chromo-LAL is reflected in the reduced rate of cleavage of the substrates and a corresponding inability of the reaction to reach maximum velocity (Vmax).

Consistent with that published in the literature, mammalian HDP LL-37, BMAP-28, SMAP-29 and CRAMP inhibited the activation of the Chromo-LAL assay at a concentration of 250 pmol/ml, indicating that they interact with LPS and thus prevent the activation of the enzymatic cascade [52], [53], [55], [61] (figure 2). BMAP-28 was the most potent, preventing the activation of the enzyme cascade with a V_max_ of 3.1 and SMAP-29 was less effective with a V_max_ of 26.5. Of the helminth-derived HDPs, both Sm16-p73 and SmHDM-1p146 displayed no LPS-binding capacity with a V_max_ of 40.4 and 31.1, respectively, which were above the control reaction with no peptide (V_max_ of 28.5). By contrast, the helminth peptide SmHDM-2p58 was the second best inhibitor of the series with a V_max_ of 12.5, just after BMAP-28.

We found that FhHDM-1p2 was capable of directly activating the Chromo-LAL assay itself (data not shown) and, therefore, its binding capacity could not be evaluated using this test. However, using a plate-binding assay we have previously demonstrated that FhHDM-1p2 does indeed bind to LPS [25].

### Helminth defence molecules do not possess antimicrobial or anti-protozoan activity

The bactericidal properties of mammalian HDPs are mediated by direct antimicrobial activities, and are therefore easily evaluated as the minimal concentration capable of inhibiting visible microbial growth (MIC) against a panel of bacterial species. Consistent with previous reports [28], [31], [62], [63], we found that SMAP-29 and BMAP-28 were effective against a broad group of gram-negative bacteria, including *E. coli*, *P. aeruginosa*, and *S. typhimurium* and two gram-positive bacteria, *S. aureus* and *S. epidermis*, with MIC values of <0.25–8 µg/ml (Table 2). LL-37 and its mouse counterpart, CRAMP, showed bactericidal activity against gram-negative bacteria with MIC ranging from 2 to 8 µg/ml, but were ineffective against the gram-positive bacteria tested (MIC<128 µg/ml). The inactivity of LL-37 against *S. aureus* and *S. epidermis* is consistent with other studies [64]. However, the anti-microbial effect of LL-37 on Staphylococcus could be strain dependent as several studies have reported an effect of this peptide on both *S epidermis* [65] and *S. aureus* [66], [67]. Despite structural similarities with the mammalian peptides tested, none of the HDMs demonstrated bactericidal activity against any species of bacteria at the concentrations tested (<0.25 to 128 µg/ml).

**Table 2:** Antibacterial activity of mammalian- and helminth-derived peptides against gram-positive and gram-negative bacteria assessed by micro broth assay.

MIC^1^ in µg/ml					
	Gram-negative			Gram-positive	
	*Escherichia coli*	*Pseudomonas aeruginosa*	*Salmonella typhimurium*	*Staphylococcus epidermis*	*Staphylococcus aureus*
FhHDM-1p2	>128	>128	>128	>128	>128
Sm16-p73	>128	>128	>128	>128	>128
SmHDM-1p146	>128	>128	>128	>128	>128
SmHDM-2p58	>128	>128	>128	>128	>128
LL-37	4	8	2	>128	>128
CRAMP	2	4	4	>128	>128
SMAP-29	<0.25	<0.25	<0.25	<0.25	2
BMAP-28	1	4	4	4	8

1 MIC: minimum inhibitory concentration determined by a conventional microbroth-based assay in Mueller Hinton medium supplemented with acetic acid/BSA [57].

**Figure 3:**
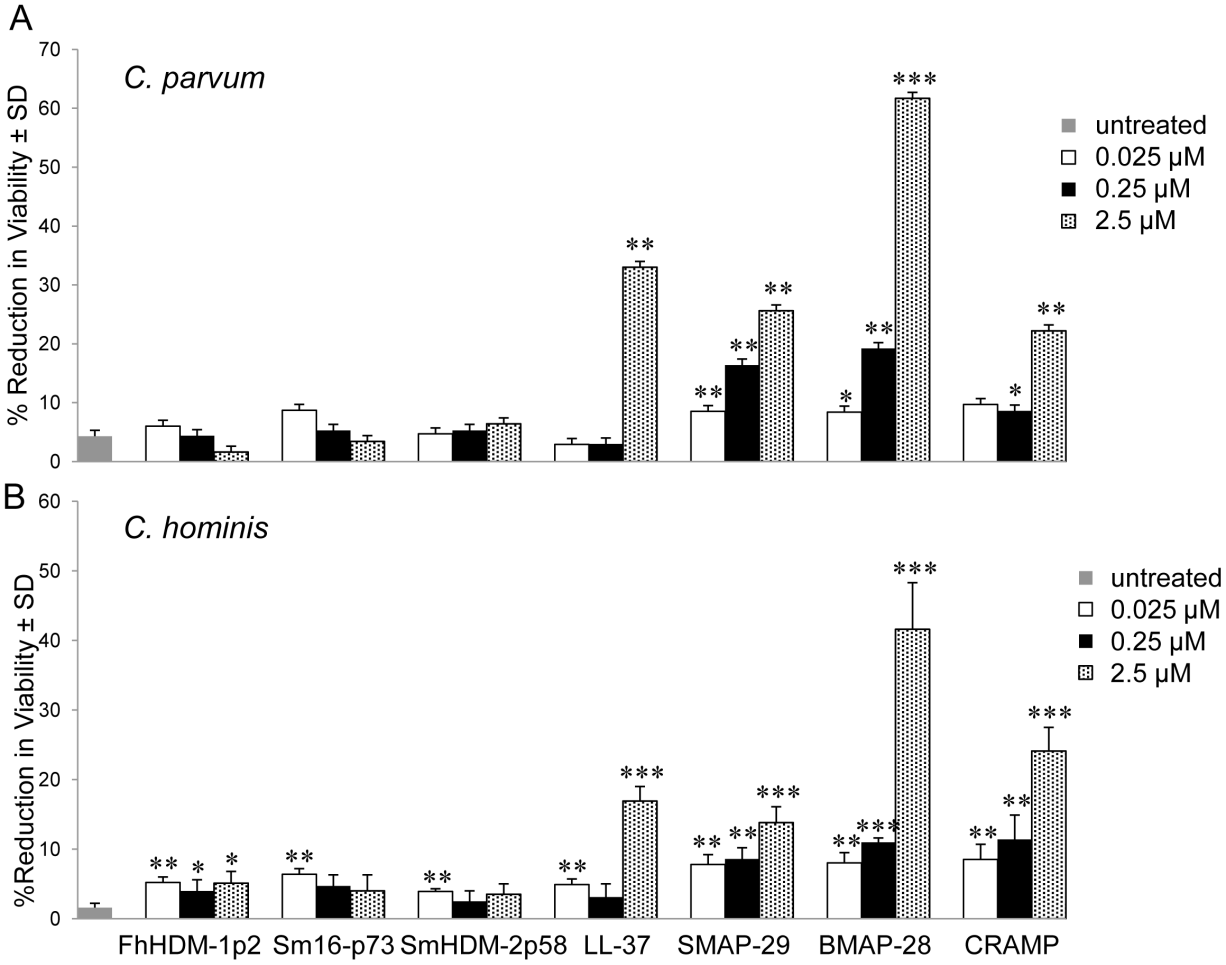
Mammalian, but not helminth, peptides reduce *Cryptosporidium spp.* sporozoite viability. The viability of *C. parvum* (A) and *C. hominis* (B) sporozoites was assessed after incubation with peptides or in medium alone (untreated). Following the addition of FDA and PI, the percent viability for each preparation was determined by epifluorescence. The data shown are means ± SD.

We have recently shown that cationic peptides, including the cathelicidin LL-37, are highly parasiticidal against the apicomplexan parasite *C. parvum in vitro* [47]. Using the same methodology, we compared the anti-parasite activity of the mammalian HDPs to that of the four helminth-derived peptides. In keeping with our previous data [35], LL-37 exhibited parasiticidal activity at a concentration of 2.5 µM against *C. parvum* and the related species *C. hominis* (figure 3). The other mammalian cathelicidins showed greater parasiticidal activity, reducing the viability of protozoans at lower concentrations of 0.025 and 0.25 µM. Of particular note, BMAP-28 demonstrated the highest potency against both species of protozoan at 2.5 µM (*P*<0.01). In stark contrast to these results, was the relative absence of parasiticidal activity of the helminth-derived peptides, even at the highest concentration of 2.5 µM.

### Helminth defence molecules are not cytotoxic to mammalian cells

**Figure 4:**
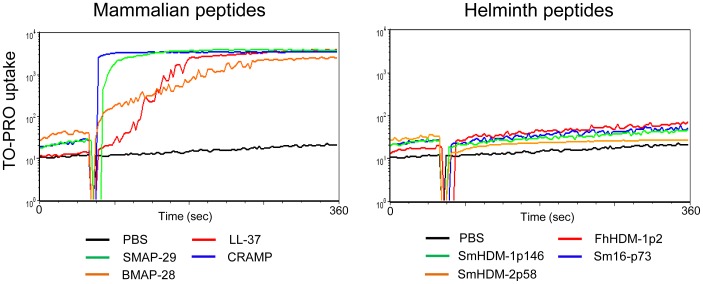
Mammalian, but not helminth, peptides induce the formation of pores in macrophages. RAW macrophages were incubated in the presence of TO-PRO. After 60 sec, 50 µM of mammalian (left panel) or helminth (right panel) peptides was added to the medium. The uptake of fluorescent dye was measured over a period of 360 sec.

The predominant mechanism of HDP bactericidal activity is the formation of pores in the membrane lipid bilayer, destroying its integrity and causing cell death [68]. However, this effect is not specific to bacterial cells and HDPs have also been shown to be cytolytic to eukaryotic cells, particularly at high concentrations [69]. TO-PRO is a membrane impermeant dye and therefore its detection within cells is indicative of pore formation. Using this dye, we demonstrated that, as expected, all mammalian peptides at a concentration of 50 µM (equivalent to the highest concentration tested in the bactericidal assays) induced the formation of pores in a murine macrophage cell line (figure 4). However, at the same concentration none of the helminth peptides exhibited this effect.

To more completely assess the cytotoxicity of the peptides, we first examined their haemolytic activity against human RBCs at various concentrations (8–256 µg/ml). After one hour of co-incubation, all of the mammalian peptides induced concentration dependent hemolysis (Table 3). BMAP-28 was the most potent of all the peptides, with 50% of RBCs lysed at the lowest concentration of peptide tested (8 µg/ml) and 70% at the highest concentration of 256 µg/ml. In comparison, at this highest concentration, the other mammalian peptides were much less cytotoxic, lysing only 14.5–29.3% of RBCs. Notably, under the same experimental conditions, the *S. mansoni*-derived peptides did not lyse the cells at any concentration tested. The *F. hepatica*-derived FhHDM-1p2 showed some low-level cytolytic activity, with 11.4% of cells lysed at the highest concentration of 256 µg/ml.

**Table 3:** Hemolytic activity of mammalian- and helminth-derived peptides.

Peptide	Hemolytic activity in % at peptide concentrations (µg/ml)					
	8	16	32	64	128	256
FhHDM-1p2	0	0	1.8	2.6	5.4	11.4
Sm16-p73	0	0	0	0	0	0
SmHDM-1p146	0	0	0	0	0	0
SmHDM-2p58	0	0	0	0	0	0
LL-37	0	1.7	4.1	10.4	20	20
CRAMP	0	2.6	5.8	9.1	20.1	29.3
SMAP-29	0	0	0	2.6	6.7	14.5
BMAP-28	50.2	60.4	48.8	49.5	52.9	70.23

**Figure 5:**
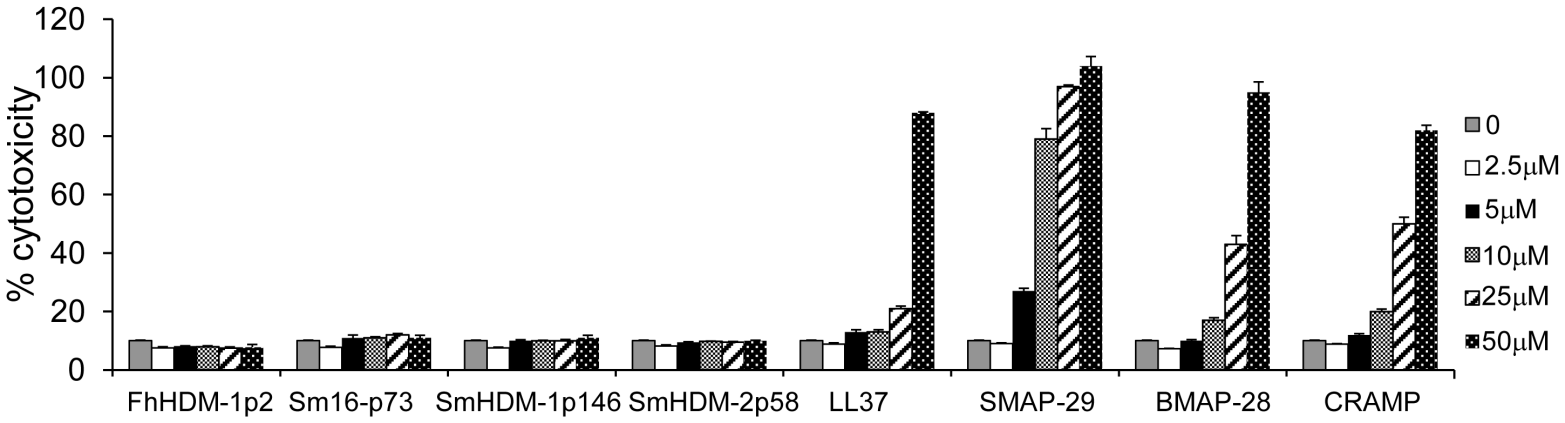
Mammalian, but not helminth, peptides induce the release of LDH from macrophages. RAW macrophages were incubated with peptides for 1 h at 37°C. The release of LDH by cells was measured and expressed as the percentage of LDH released after treatment of cells with lysis buffer (regarded as 100%). Data shown are the means ± SEM of triplicate samples and representative of three independent experiments.

Lactate Dehydrogenase (LDH) is a soluble cytosolic enzyme that is released into culture media following loss of membrane integrity resulting from either apoptosis or necrosis. Therefore LDH release is an indicator of cell membrane integrity and acts as a measure to assess cytotoxicity. Consistent with the demonstration of hemolysis, higher concentrations (>10 µM) of mammalian peptides also resulted in the death of murine macrophages, with the highest concentration tested (50 µM) resulting in 100% cytotoxicity, compared to the effect of a lysis buffer (figure 5). By contrast, none of the helminth peptides induced cell death at any concentration tested. This lack of LDH detection was not due to enzyme inhibition by the helminth peptides; when the peptides were added directly to culture supernatant from lysed cells the LDH activity was unchanged. Consistent with these data, the cells treated with helminth peptides (10–50 µM) looked morphologically normal by light microscopy (data not shown).

### Both mammalian- and helminth-derived peptides suppress secretion of the inflammatory cytokine TNF from macrophages

**Figure 6:**
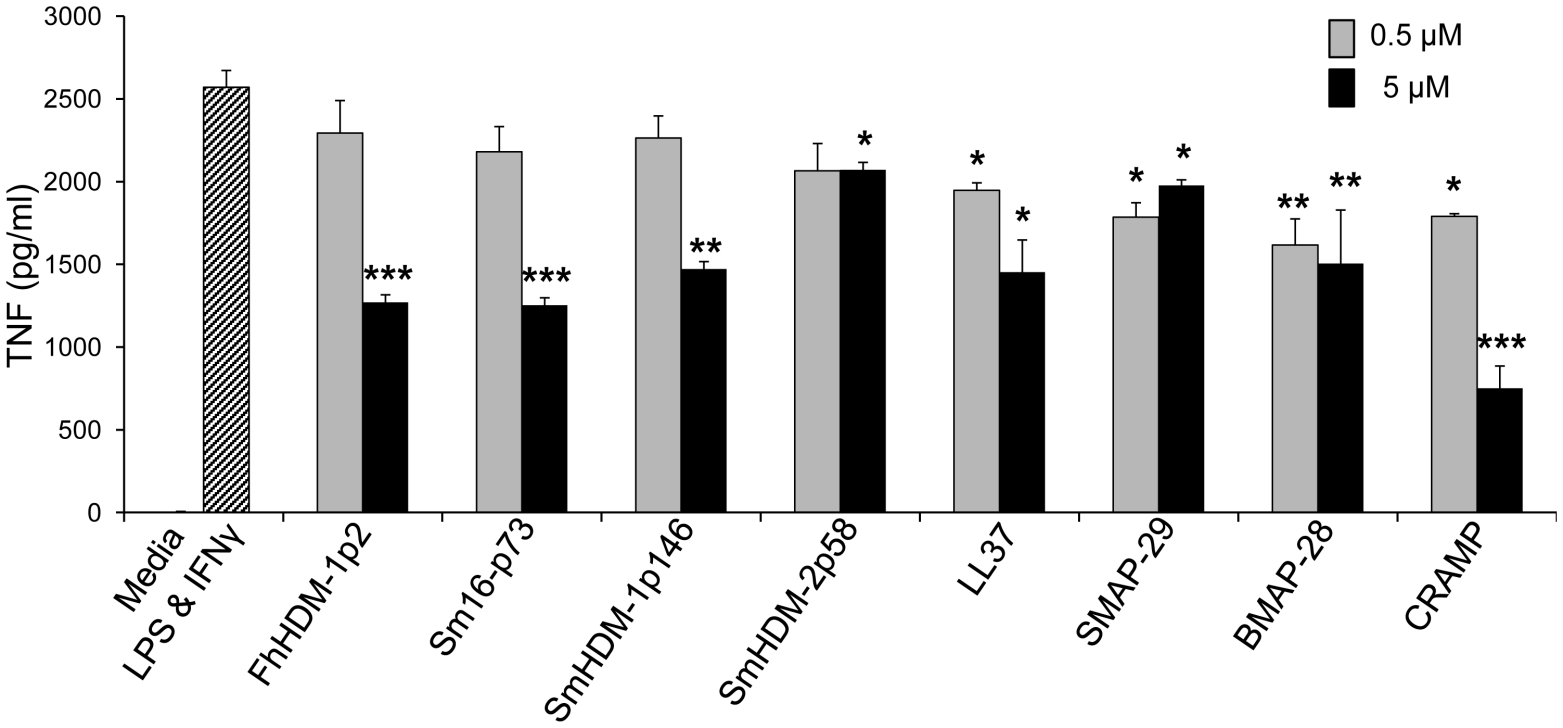
Both mammalian and helminth peptides suppress secretion of the inflammatory cytokine TNF from macrophages. Primary murine bone marrow derived macrophages were treated with peptides for 1 h, washed then subsequently stimulated with a combination of *E. coli* LPS (10 ng/ml) and IFNγ (10 ng/ml) for 16 h. The amount of TNF (pg/ml) secreted into the culture media was measured by ELISA. The data shown are means ± SEM of triplicate samples and are representative of three independent experiments.

Activation of macrophages by microbial stimuli is central to the induction of innate immune responses. IFNγ is one of the key cytokines in the innate immune response to intracellular pathogens, and augments cellular responses to TLR ligands such as bacterial LPS [70], [71]. To prevent excessive inflammation potentially leading to sepsis, HDPs have been shown to inhibit the response of macrophages to these inflammatory mediators using mechanisms that are independent of direct binding to LPS [72]. Significantly, macrophages isolated from animals and humans infected with helminth parasites are also hyporesponsive to stimulation with LPS and IFNγ [73], [74]. Therefore, here we investigated whether helminth-derived peptides, like mammalian HDPs, could suppress the activation of inflammatory macrophages. At concentrations below cytotoxic levels (<5 µM), all mammalian peptides significantly inhibited TNF production in response to the combined stimulation with LPS and IFNγ (figure 6). Titration of the peptide concentration showed that even at concentrations as low as 0.5 µM, the inhibitory activity was preserved. In contrast, at the lowest concentration tested, the helminth-derived peptides had no effect on the activation of macrophages. However, as the concentration was increased, the helminth peptides significantly suppressed the inflammatory response of macrophages and in most cases more effectively than the mammalian peptides (figure 6). It is worth noting that the helminth-derived peptides could be tested at concentrations up to 50 µM as they are non-toxic to cells, whereas due to their cytotoxicity the HDPs were not tested at concentrations above 10 µM (figures 4,5). At these higher concentrations (10, 25 and 50 µM) the helminth-derived peptides significantly reduced the production of TNF from activated macrophages in a concentration dependent manner (data not shown).

### Both mammalian- and helminth-derived peptides alter the secretion of immunoglobulin from activated B cells

**Figure 7:**
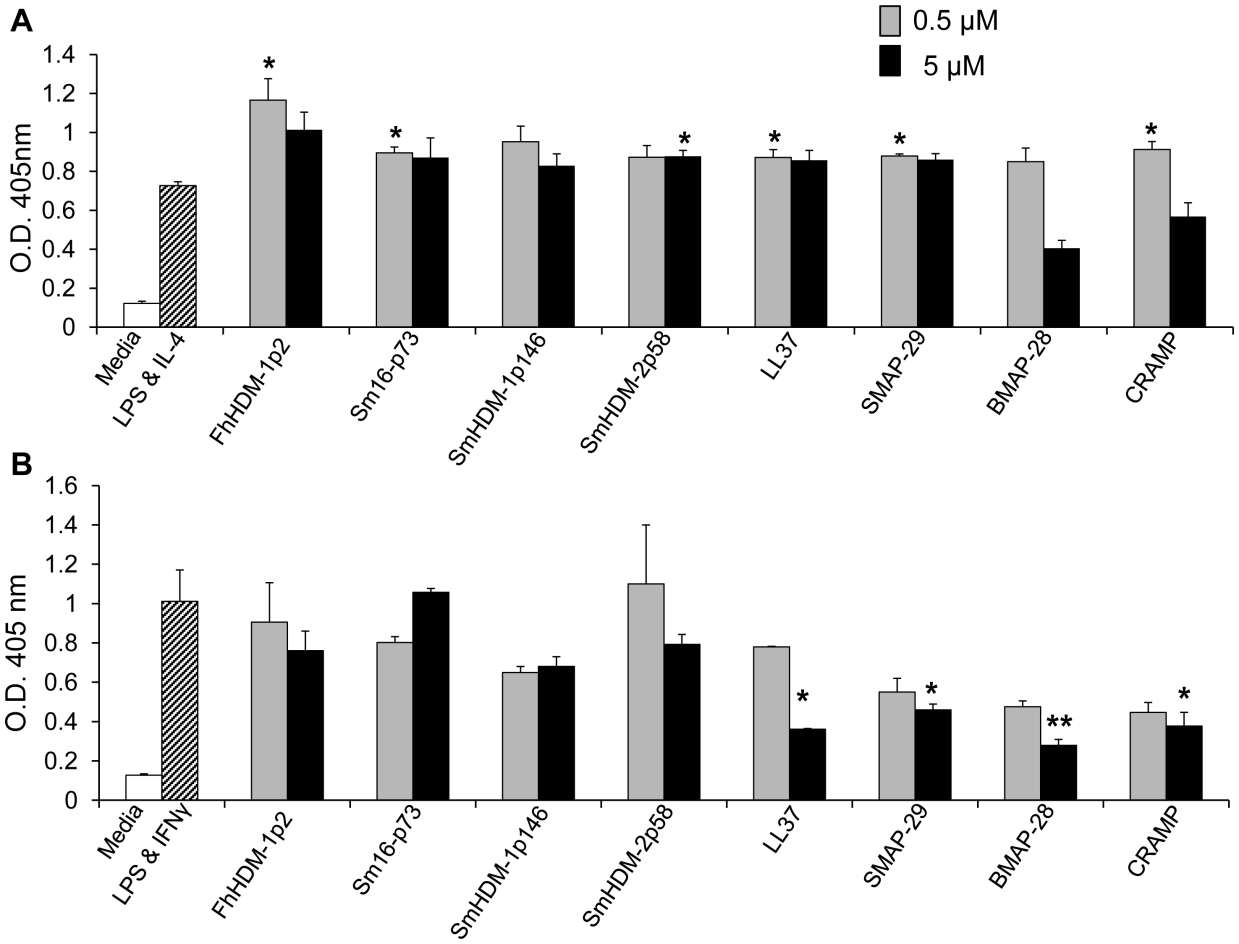
Both mammalian and helminth peptides alter the secretion of immunoglobulin from activated B cells. Murine splenic B cells were treated with a range of concentrations of peptides for 1 h at 37°C, washed and then incubated with a combination of either (A) *E. coli* LPS (10 µg/ml) and IL-4 (10 ng/ml) or (B) *E. coli* LPS (10 µg/ml) and IFNγ (200 ng/ml) for six days. Levels of IgG1 (A) and IgG2a (B) in cell supernatants were measured by ELISA. The data shown are from triplicate samples, corresponds to the means ± SEM, and are representative of two independent experiments.

In addition to directly inhibiting inflammatory innate immune responses, there is evidence that mammalian HDPs have an additional role in regulating the magnitude of the adaptive antibody responses. For example, it has been shown that CRAMP functions to positively regulate the level of IgG1 produced by B cells [75], and LL-37 reportedly decreased the production of IgG2a from mouse splenic B cells activated with LPS and IFNγ [76]. Consistent with these reports, our analyses showed that with the exception of BMAP-28, all the mammalian HDPs significantly increased the production of IgG1 in response to a Th2 biased environment (LPS and IL-4) (figure 7A). The apparent reduction in IgG1 production recorded for the higher concentration of BMAP-28, likely reflects some level of cell death rather than a reduction in antibody production. Due to this cytotoxicity the HDPs were not tested at concentrations above 5 µM. While there was greater variability between the HDMs, peptides from *F. hepatica* and *S. mansoni* significantly increased the production of IgG1 in response to LPS and IL-4 even at low concentrations.

Conversely, mammalian peptides reduced the production of IgG2a in a Th1 (LPS and IFNγ) biased environment (figure 7B). For the helminth peptides, only concentrations above 5 µM had the same effect on B cells, significantly (*p*<0.001) inhibiting IgG2a secretion (data not shown), suggesting a lower potency than mammalian-derived peptides.

## Discussion

Parasitic helminths secrete molecules that modulate host immune responses to establish an environment that facilitates their survival and a prolonged reproductive phase [20], [77], [78]. Co-evolution of helminths with their hosts means that these parasites are well adapted to the host's immune system, making use of endogenous regulation mechanisms to manipulate the immune response to their benefit. In this study, we compared the biological activities of a series of helminth-derived cathelicidin-like peptides to that of their mammalian homologues and suggest how their production by helminths can facilitate a successful parasitic life cycle.

*In vitro*, most mammalian HDPs are effective antimicrobial agents against a range of organisms including gram-negative and gram-positive bacteria, protozoa, viruses and fungi [10], [79], [80]. In general, the expression of HDPs is increased at the onset of an infection and therefore the anti-pathogenic activity was thought to be one of the most important immediate responses that the mammalian host evolved to deal with invading pathogens. It has been proposed that the specificity of HDPs for particular microbes is subjected to significant variation and is particularly influenced by the types of microbial biotas to which each HDP species is exposed [81]. Consistent with this theory, we showed some variation in the anti-microbial capabilities of the mammalian HDPs examined. While sheep and bovine derived peptides were effective against a broad range of both gram-positive and gram-negative bacteria, the mouse and human HDPs were largely ineffective against the gram-positive species tested. However, we found that none of the helminth-derived peptides displayed gram positive or negative bactericidal activity, even at the highest concentration tested, implying that their specialised function is not anti-microbial. However, we cannot exclude the possibility of the peptides having anti-microbial activity on other, untested pathogenic bacteria.

Despite lacking bactericidal activity, we showed previously that FhHDM1-p2, like the mammalian peptides, interacts with LPS, thus effectively neutralising the ability of infecting bacteria to induce an inflammatory response [25]. We suggested that this may be a mechanism used by the parasite to prevent excessive activation of innate cells in response to the translocation of microbes into circulation occurring as a result of damage to the skin and/or gut epithelium during migration of the parasite [25]. As the two major factors mediating interaction between LPS and HDPs are hydrophobicity and cationicity [82], inspection of the sequence of the other HDMs would predict a universal ability to bind to LPS. However, while SmHDM-1p146 appeared to bind LPS as efficiently as the mammalian peptides, neither SmHDM-2p58 or Sm16-p73 were particularly potent, indicating that the neutralization of LPS may not be a common function of the helminth-derived peptides.

A number of studies have demonstrated the ability of amphibian and mammalian HDPs to kill protozoan parasites *in vitro* [83], [84]. For example, BMAP-28 possesses potent activity against the agent of human leishmaniasis, *Leishmania major* [83], and we have shown that LL-37 can reduce the viability and infectivity of sporozoites of *C. parvum*, an intestinal infection of humans and agricultural animals [47]. In the present study we confirm the activity of LL-37 against *C. parvum* and the related parasite *C. hominis* and show that the other mammalian cathelicidins tested also have anti-protozoan activity; BMAP-28 exhibited the most potent *in vitro* activity. By contrast, and consistent with their lack of antibacterial activity, the helminth-derived HDMs did not kill either parasite *in vitro*. Clearly, the particular physico-chemical properties of HDMs do not confer an ability to penetrate and disrupt the surface membrane of these parasitic organisms.

Although widely defined as antimicrobial, in fact, at the concentrations normally found at human mucosal surfaces and in physiological salt conditions, the mammalian cathelicidin-like peptides do not display bactericidal activity. However, at these same concentrations and under the same conditions, the peptides exhibit a variety of immune modulatory functions [12], [85]. This has led to the suggestion that the cathelicidins are principally immune modulators rather than antimicrobials, and like the mammalian defensins, have traded their bactericidal capacities to acquire the ability to broadly regulate the immune response [12], [86]. The mammalian cathelicidins have a diverse effect on the cellular immune response, but in particular, the peptides have a crucial role in regulating TLR-dependent innate inflammatory responses. This means that they function to maintain homeostasis in response to natural shedding of microflora-TLR agonists as well as controlling the systemic inflammatory response to infection or tissue damage [12], [86], [87]. We have shown here that the helminth-derived cathelicidin-like peptides also regulated the innate immune response to TLR stimulation by inhibiting TNF release from macrophages stimulated with bacterial LPS. While the helminth HDMs were not as effective as their mammalian homologues at the lowest concentration tested, they were correspondingly more potent as their concentration was increased towards quantities that are likely secreted during infection. It is probable that the function of these secreted HDMs is similar to the predicted role for the mammalian HDPs, i.e. prevention of an excessive inflammatory response, which acts to prevent the expulsion of the worm and to protect the host from exacerbated tissue damage.

The role of HDPs in regulating the adaptive immune response has been less extensively studied. Recent studies have shown that LL-37 decreased the production of IgG2a from murine B cells stimulated with LPS and IFNγ [76], and that CRAMP increased the amount of IgG1 in response to IL-4 [75]. These results are consistent with the suggestion that HDPs are engaged in the process of infection resolution and wound healing, as autoreactive IgG1 antibody production is central to tissue repair processes [88], while IgG2a are associated with IFNγ/Th1-mediated inflammatory responses. Consistent with these reports, the helminth-derived peptides performed in a similar manner to their mammalian counterparts, successfully enhancing the production of IgG1. At high concentrations, the helminth-derived peptides were far more effective than mammalian HDMs at suppressing the release of IgG2a in response to IFNγ, supporting their role as potent anti-inflammatory agents.

Due to their immune modulatory activities, there is considerable interest in developing HDPs as therapeutics such as anti-inflammatory agents, adjuvants and wound healing agents. The therapeutic potential of HDPs has been demonstrated and a number of peptides are being developed as anti-inflammatory agents [89]. However, the clinical use of these peptides as injectable therapeutics has been hampered by indications of toxic side-effects on mammalian cells and their ability to lyse eukaryotic cells [90], [91]. This had led to intense research into understanding how HDPs function in terms of their physico-chemical properties. Among the factors that appear to influence specificity between their activity against prokaryotic and eukaryotic cells are the ability to form an amphipathic α-helical structure, hydrophobicity, overall charge distribution, and minimal peptide length [92]. At first sight, the biochemical characteristics of the helminth-derived HDMs would predict an inherent cytotoxic activity: they form amphipathic helices (figure 1), they have a comparable proportion of hydrophobic amino acids to mammalian HDPs and most of them are cationic (Table 1). However, based on the assays employed in this study, we found no correlation between the level of hydrophobicity and cytotoxic activity.

The majority of mammalian HDPs have an overall net charge ranging from +4 to +6.37 [93], implying an optimal range for biological activity. HDPs with a net positive charge of <+4 are found to be inactive, whereas increasing the net charge from +4 to +8 confers antimicrobial activity and some haemolytic activity [79]. Three of the HDMs used in this study, FhHDM-1p2, SmHDM-1p146 and SmHDM-2p58, all had a net charge <+4 which is consistent with a non-bactericidal, non-haemolytic peptide. However, despite possessing a net charge of +5, Sm16-p73 displayed neither antimicrobial nor cytotoxic activities. Recent studies have proposed that rather than a simple correlation between net charge and haemolysis it is the localisation of the positively charged amino acids within the peptide that also dictates membrane interaction and selectivity. By increasing the charge of an amphipathic HDP analog from +8 to +9, by the addition of one positive charge on the polar face, the haemolytic activity of the peptide was enhanced 32-fold [79]. Using this parameter, we calculated that Sm16 has four positively charged amino acids on its polar face compared to six for LL-37, CRAMP and BMAP-28 and seven for SMAP-29, which may provide some clue as to the difference in cytotoxicity between these peptides. Likewise, all the helminth HDMs used in this study have less positive charges on their polar face compared to LL-37, which was the least cytotoxic of all the mammalian peptides examined in this study.

It is generally accepted that the cytoplasmic membrane is the main target of many mammalian HDPs, whereby peptide accumulation in the membrane causes increased permeability and a loss of barrier function, resulting in the leakage of cytoplasmic components and cell death [94]. However, the cytotoxic concentrations of HDPs are higher than the concentrations required for the destruction of microbes, which, some authors suggest, reveals a cell-selective killing mechanism [85], [94]. The physiological concentration of HDPs at mucosal sites is typically less than 2 µg/ml [12], [80], well below the concentration that is cytotoxic to mammalian cells *in vitro*. We have found that helminth HDMs are abundant molecules within the secretions of helminth parasites, which during a multi-parasite infection would likely be at relatively high concentrations in circulation. Therefore, it is essential for the success of the parasite that these peptides do not possess cytotoxic activity, whilst at the same time retain the beneficial immune modulatory properties. The complete absence of antimicrobial activity by helminth-derived peptides is likely linked to this need to prevent host cell death during infection.

The extraordinary capacity of helminths to regulate the immune response is central to their longevity in the mammalian host and thus underpins their success as parasitic organisms [77], [78]. Therefore, it is perhaps unsurprising that helminth secretory products contain homologues of components of the host immune system that target the same mammalian pathways. In addition to the HDMs identified here, helminth parasites express highly conserved cytokine gene families that, like their mammalian counterparts, ligate specific receptors on immune cells. *Brugia malayi* and *Ancylostoma ceylanicum* express homologues of the mammalian cytokine macrophage migration inhibitory factor (MIF) [95], and in a Th2 environment, such as that activated by helminth infection, *Brugia* MIF synergises with IL-4 to induce the development of regulatory M2 macrophages [96]. Helminths also express members of the Tumour Growth Factor-(TGF)β and TGF-β receptor superfamilies [97]–[99], and similar to the mammalian cytokine, *Heligmosomoides polygyrus* TGF-β homologue has been shown to directly induce the differentiation of regulatory T cells, demonstrating a key role in parasite immune regulation [100].

The cysteine protease inhibitors, cystatins, are an ancient and conserved family of peptides in the animal and plant kingdoms [101]. The cystatins of parasitic worms differ substantially from those produced by free-living nematodes with regard to their immune modulatory properties [102]. In particular, the acquisition of an asparaginyl endopeptidase site, similar to that of vertebrate cystatin C, confers an ability to reduce the activation of host T cell responses by directly inhibiting the presentation of antigen by dendritic cells [103], [104], suggesting a specific adaption to regulate host immune responses [102], [105]. Similar to the cystatins, HDPs are conserved in all organisms, including plants, animals and humans [106]. However, we show here that while the helminth-derived HDMs are effective immune modulators, they display no bactericidal activity. These observations would suggest that like the cystatins, HDMs have become specifically adapted to support a parasitic lifestyle, losing the more ancient property of direct antimicrobial killing but acquiring the ability to regulate immune responses in order to promote their survival within the mammalian host. It is possible that HDM immune modulation arose in trematodes following their divergence from the chordate lineage (as acoelomates) and their subsequent specialisation to an endoparasitic lifestyle, distinct from the free-living acoelomate turbellarian flatworms from which HDM homologues have yet to be identified (unpublished observation).

The immune-modulatory properties of mammalian HDPs, and in particular their ability to prevent excessive inflammatory induced pathology associated with bacterial sepsis, has attracted interest in exploiting these as anti-infectives. However, their cytotoxicity, as also shown in the present study, has presented a major drawback for their *in vivo* use. Accordingly, the absence of cytotoxicity and retention of immune-modulatory activity observed for the helminth-derived HDMs offer an opportunity to design novel immunotherapeutics to combat microbial pathogens and immune-related disorders.
